# Supplementary figures and images for: Plant autophagosomes mature into amphisomes prior to their delivery to the central vacuole
Source: J Cell Biol. 2022 Oct 19;221(12):e202203139. doi: 10.1083/jcb.202203139 (PMC9584626; doi:10.1083/jcb.202203139)

# mCherry-CFS1

**A**

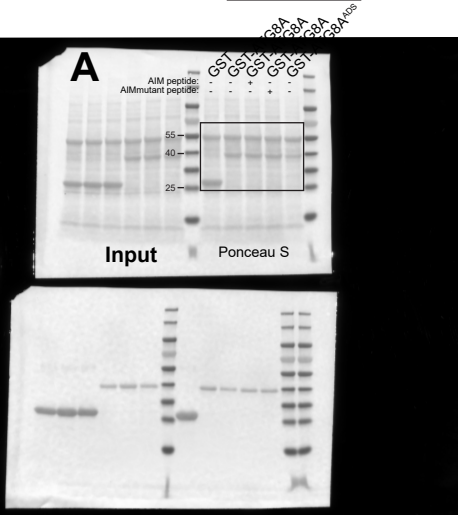

# mCherry-CFS1

**A**

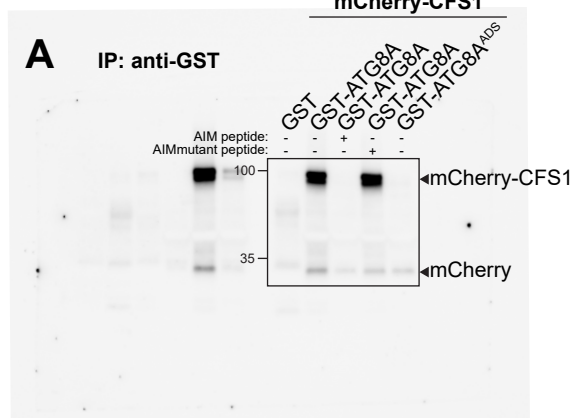

**A**

Input

# mCherry-CFS1

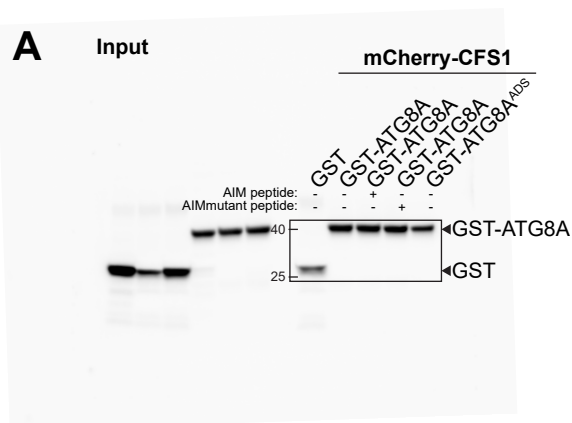

**A**

IP: anti-GST

# mCherry-CFS1

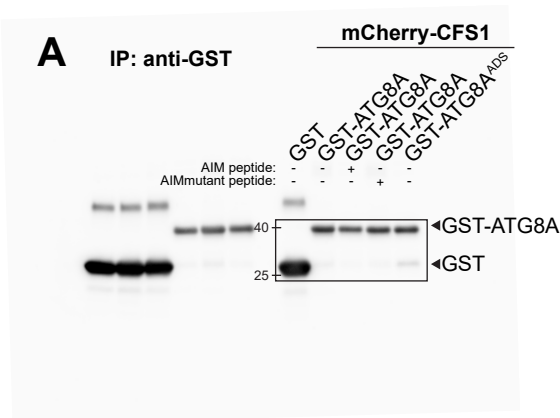

# mCherry-CFS1

**A**

Input

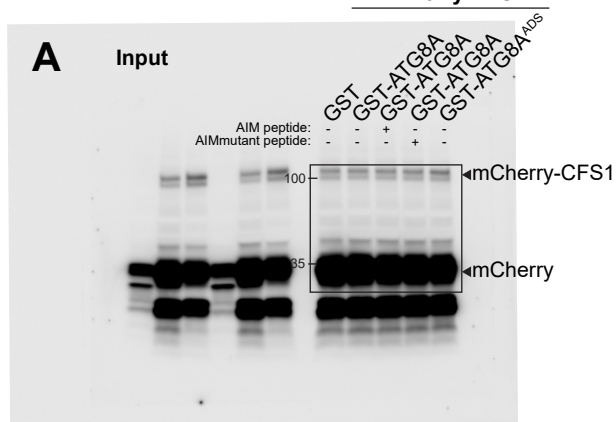

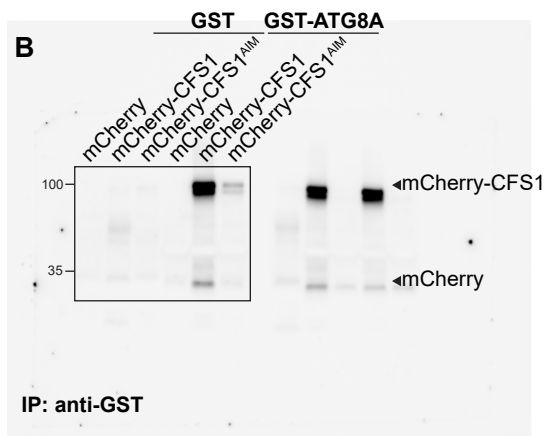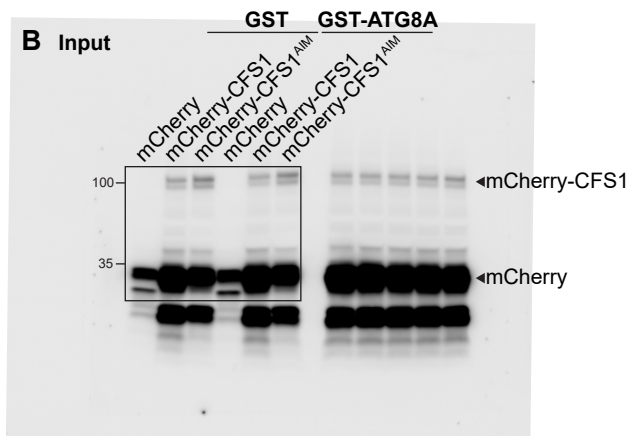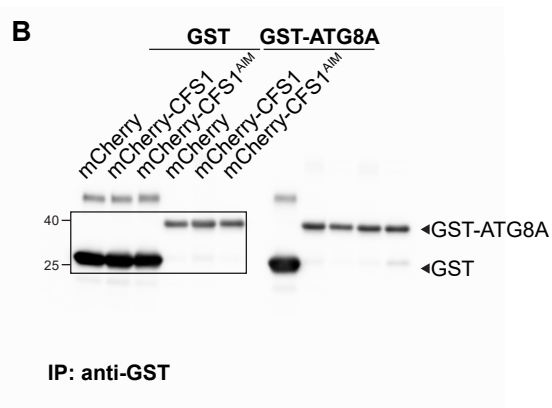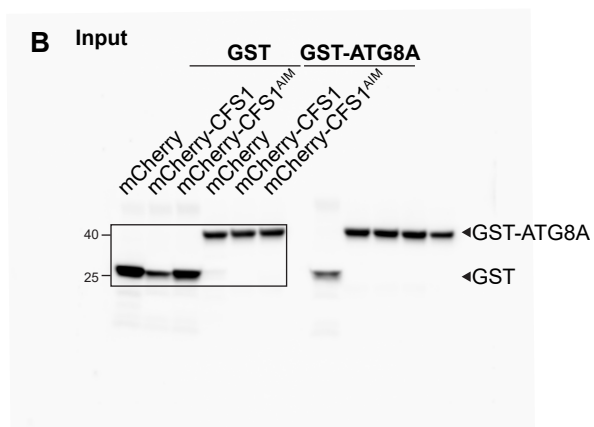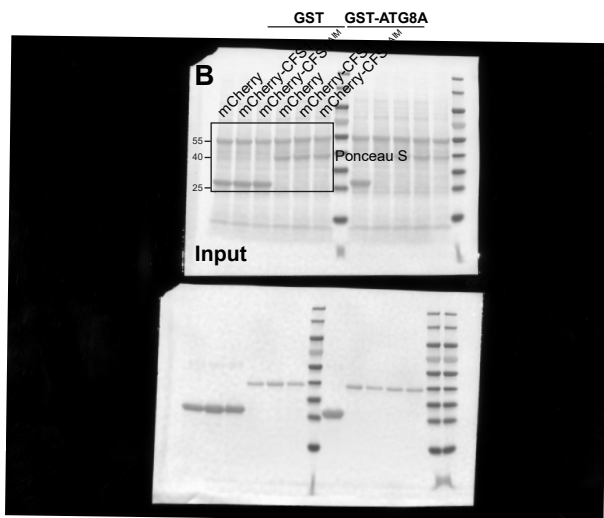

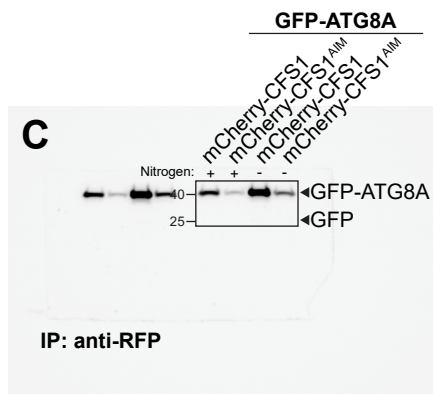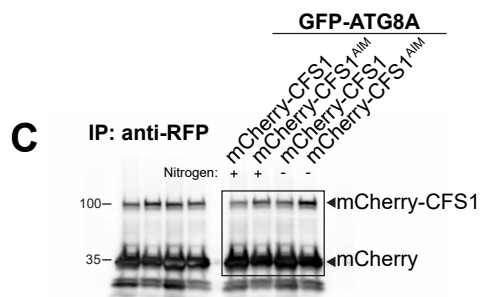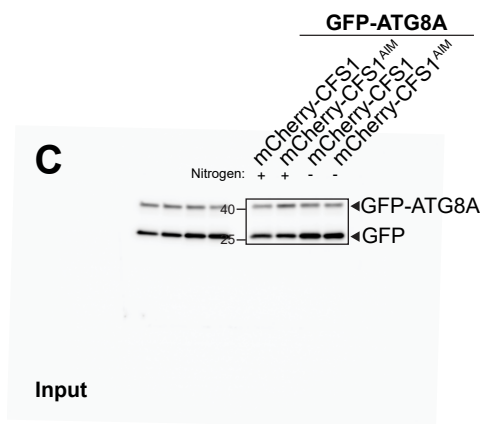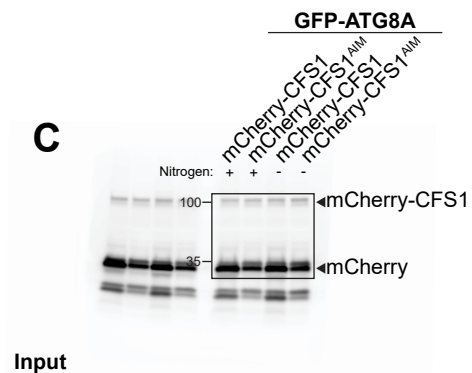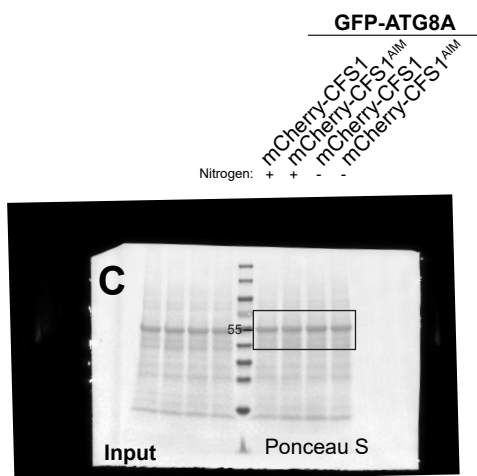

Supplement: SourceData F2 — is the source file for Fig. 2. [file JCB_202203139_SourceDataF2.pdf]

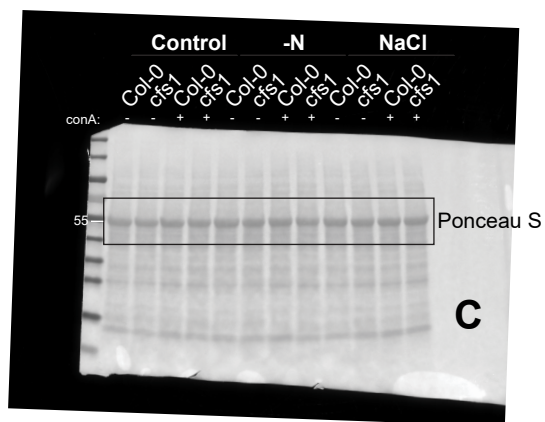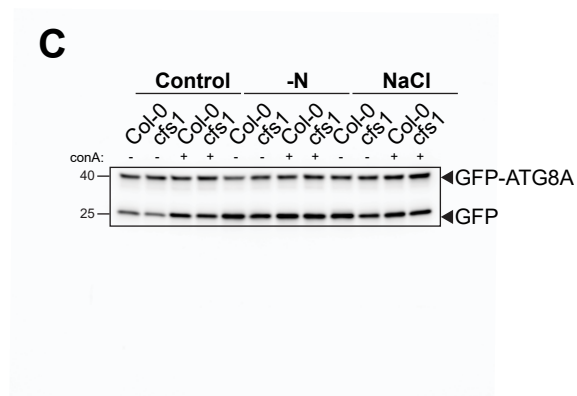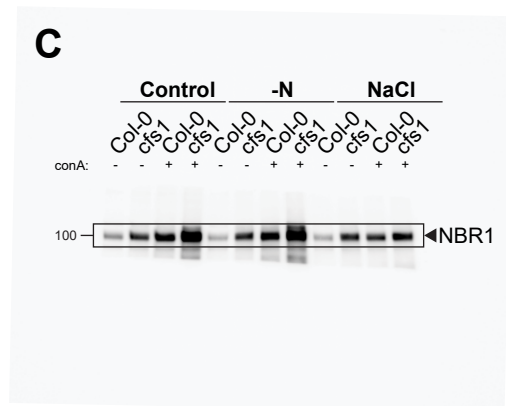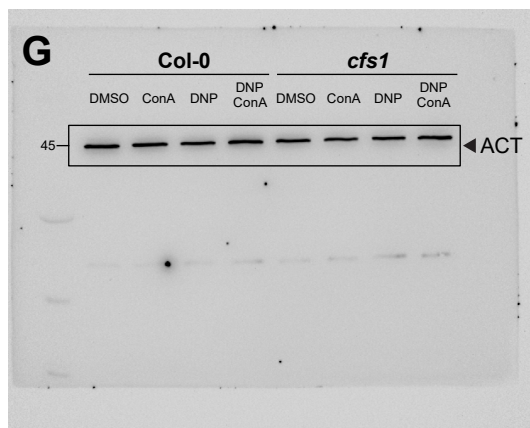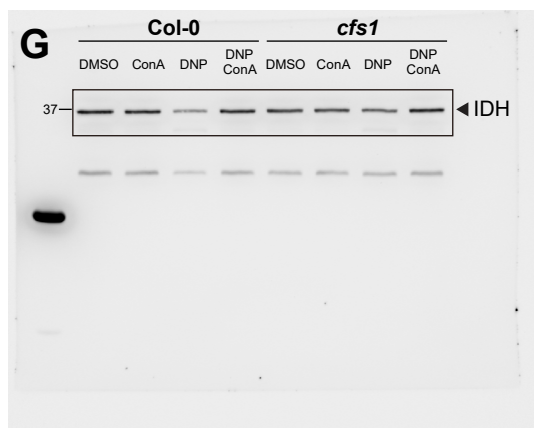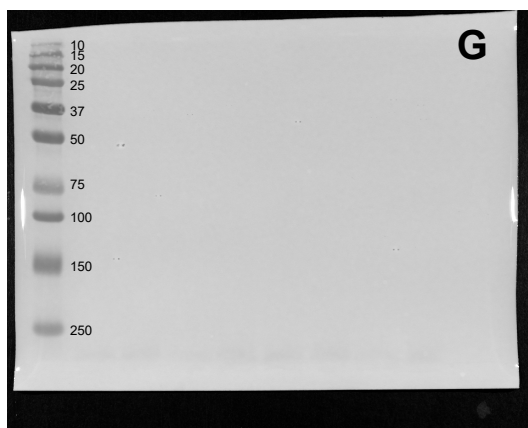

Supplement: SourceData F4 — is the source file for Fig. 4. [file JCB_202203139_SourceDataF4.pdf]

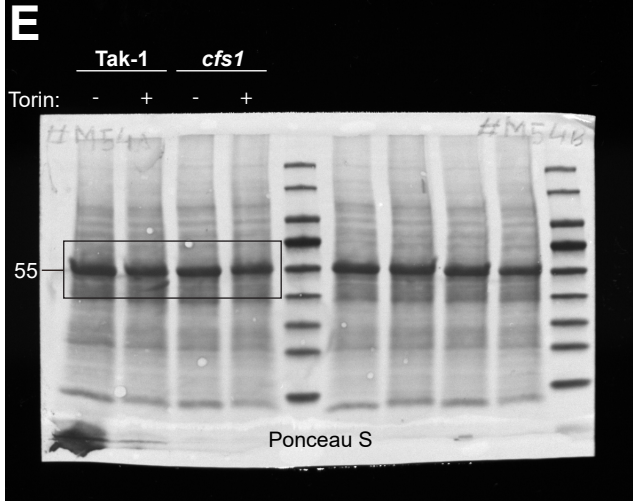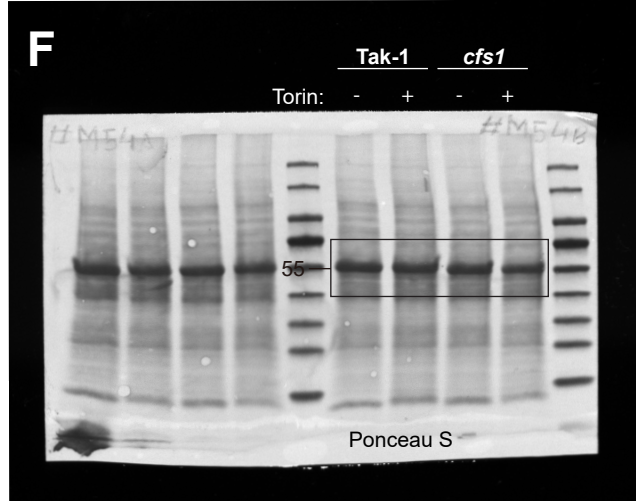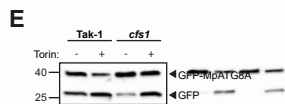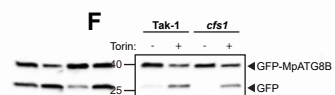

Supplement: SourceData F5 — is the source file for Fig. 5. [file JCB_202203139_SourceDataF5.pdf]

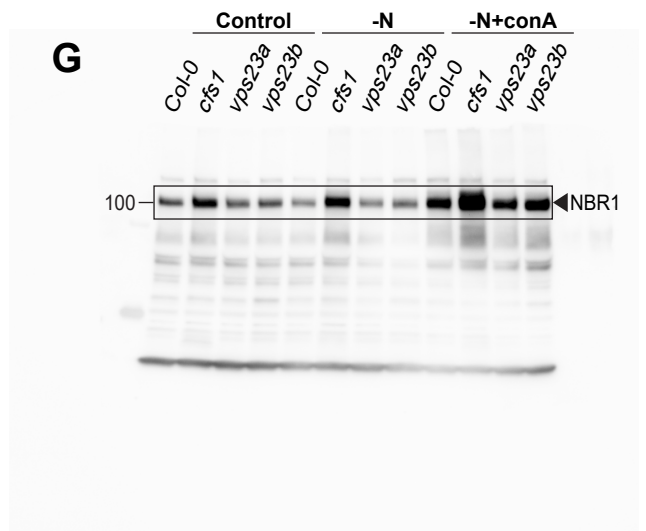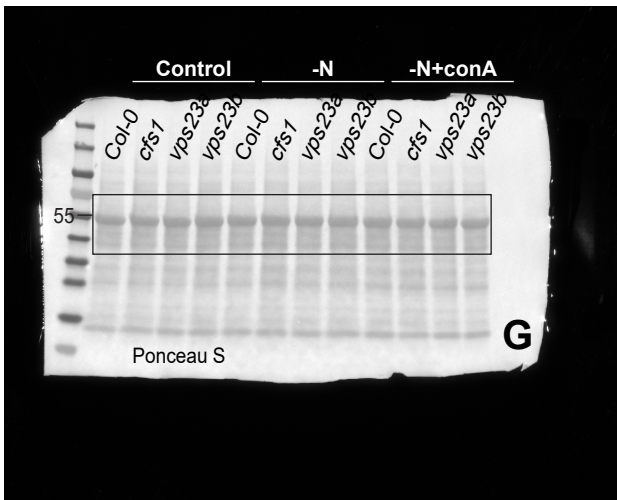

Supplement: SourceData F7 — is the source file for Fig. 7. [file JCB_202203139_SourceDataF7.pdf]

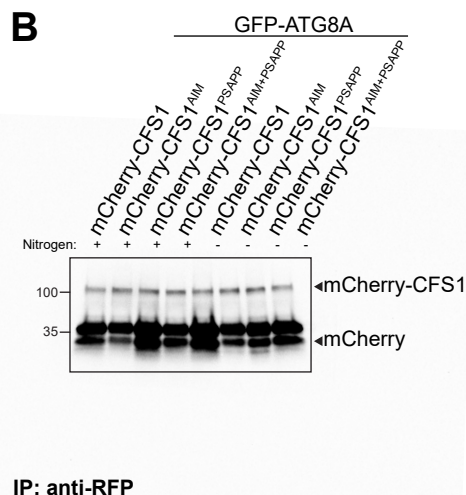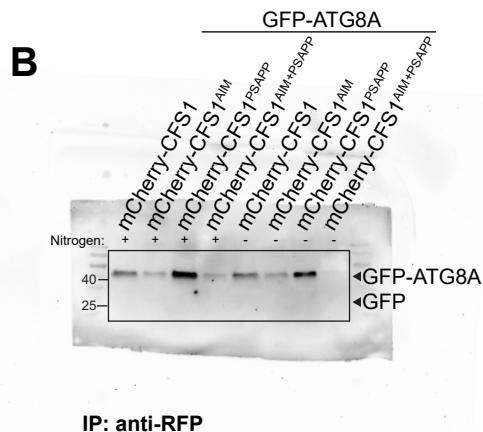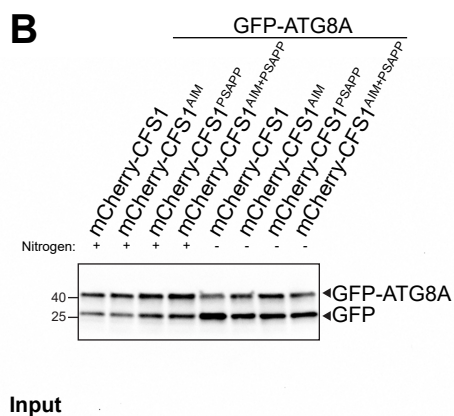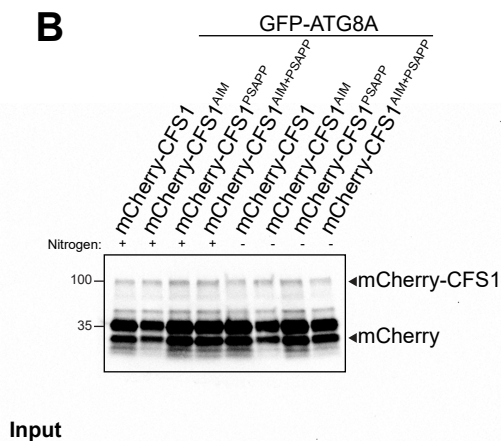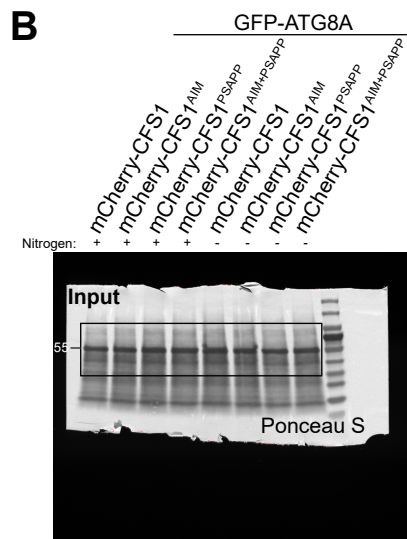

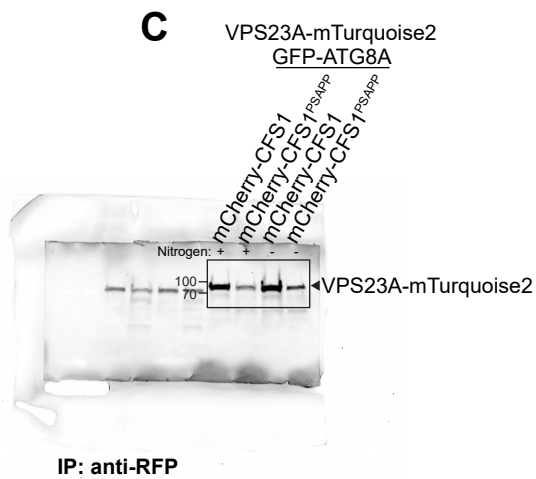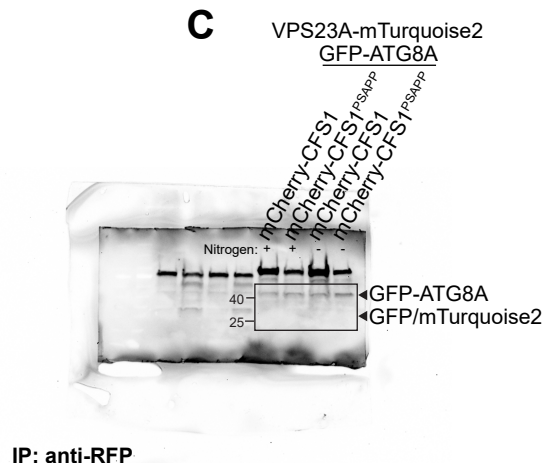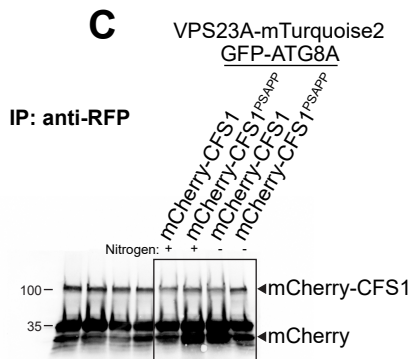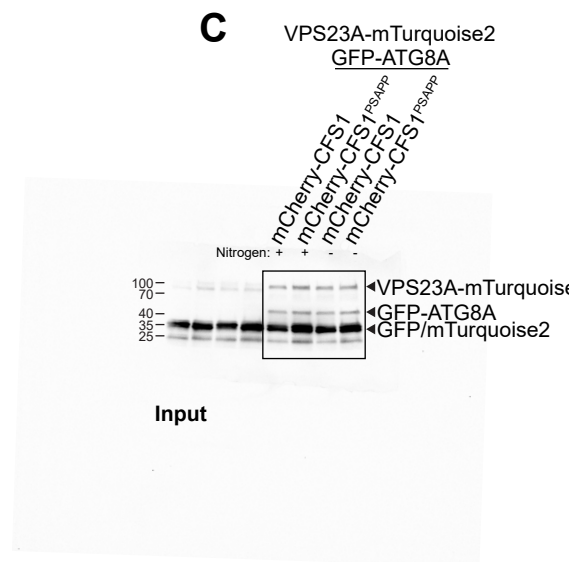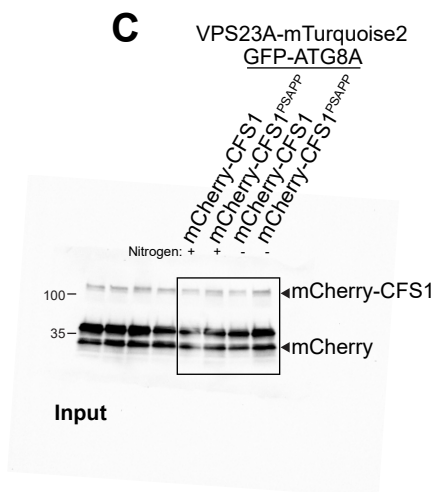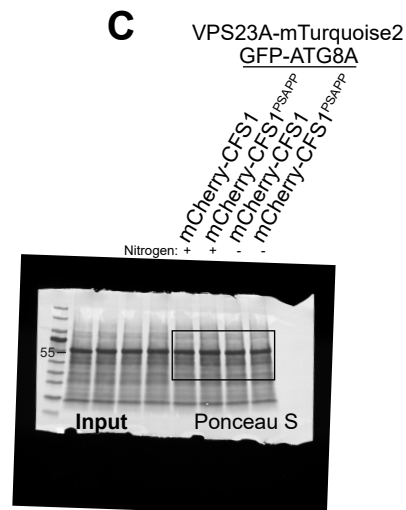

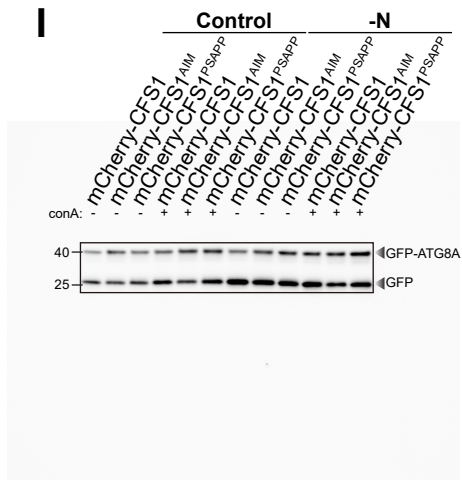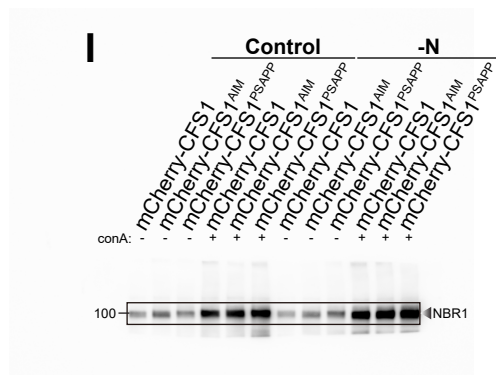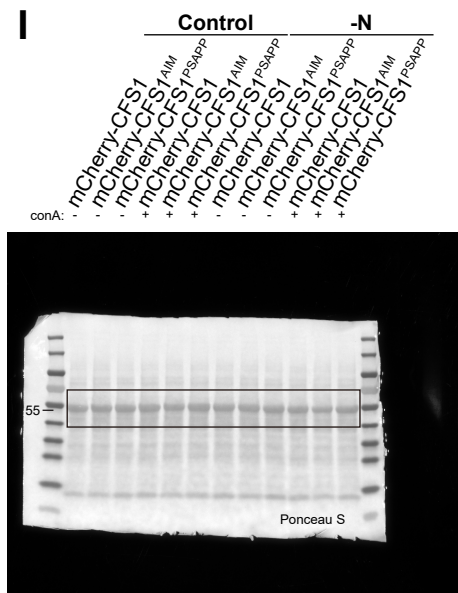

Supplement: SourceData F8 — is the source file for Fig. 8. [file JCB_202203139_SourceDataF8.pdf]

**A**

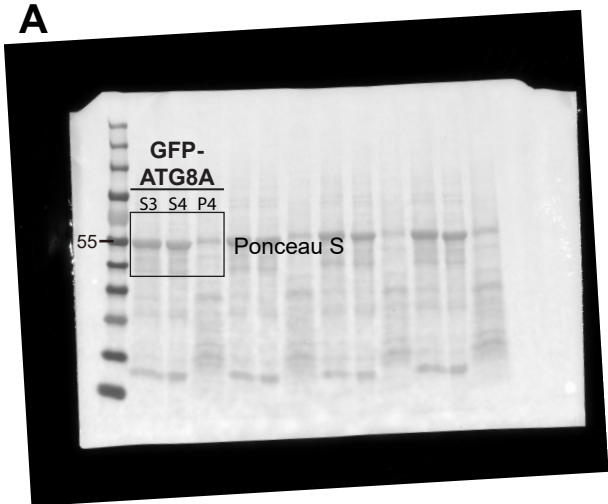

**A**

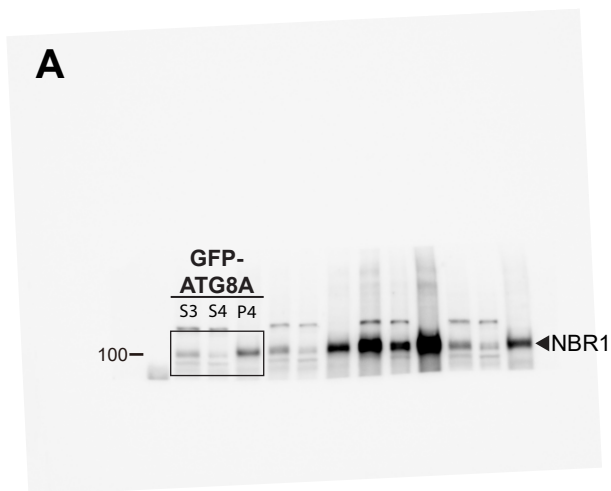

**A**

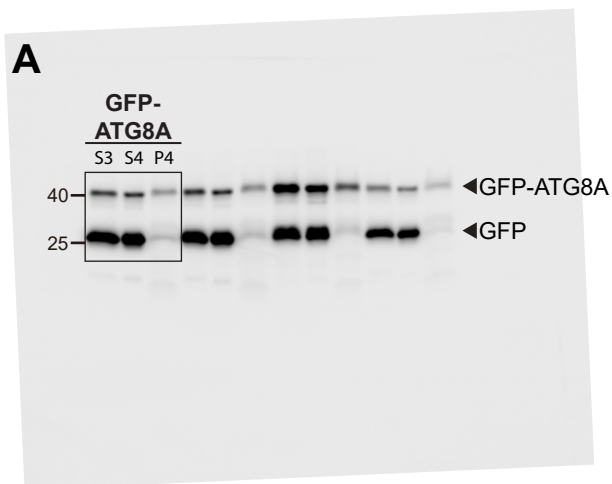

**B**

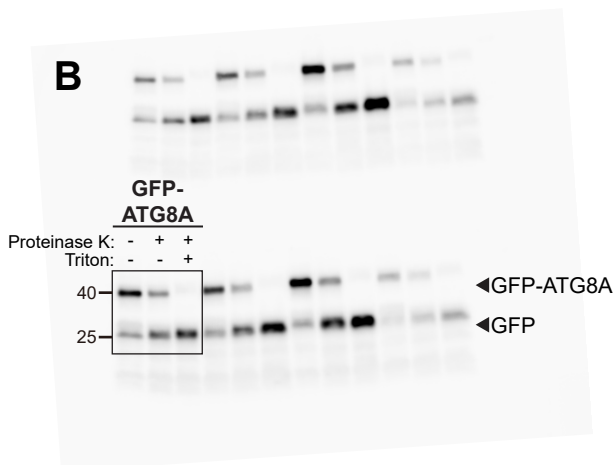

**B**

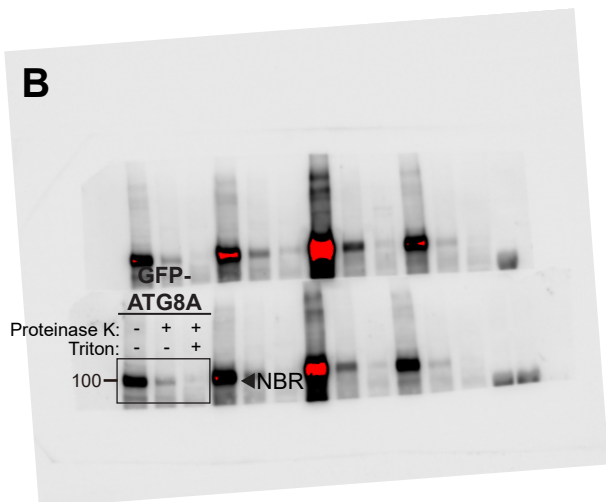

**B**

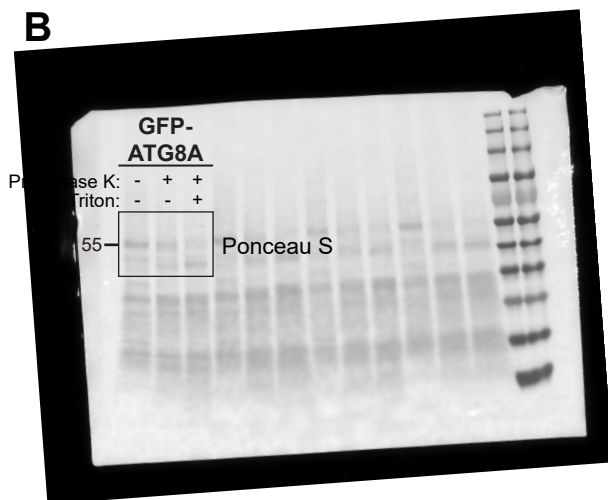

Supplement: SourceData FS1 — is the source file for Fig. S1. [file JCB_202203139_SourceDataFS1.pdf]

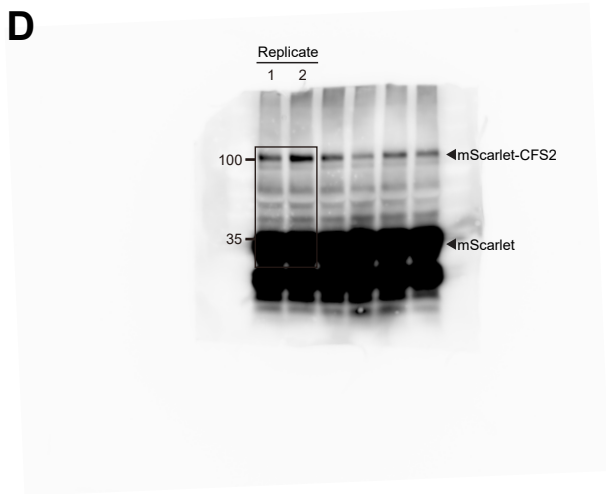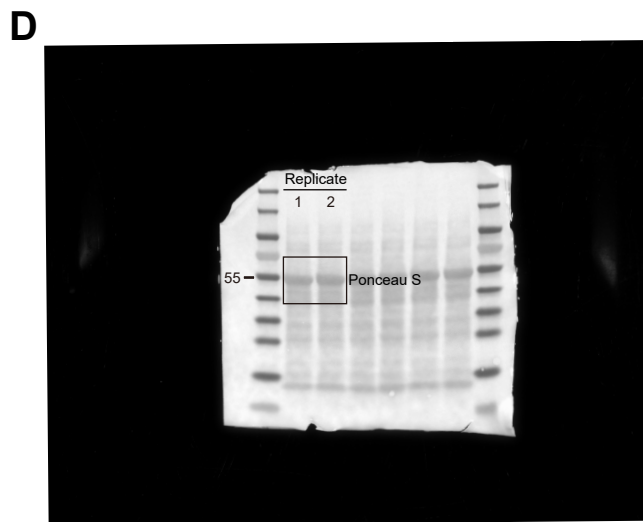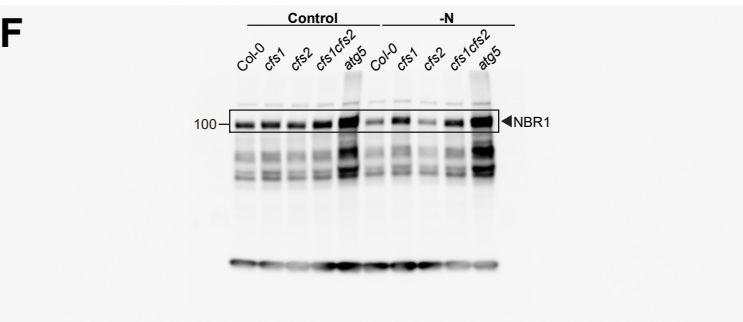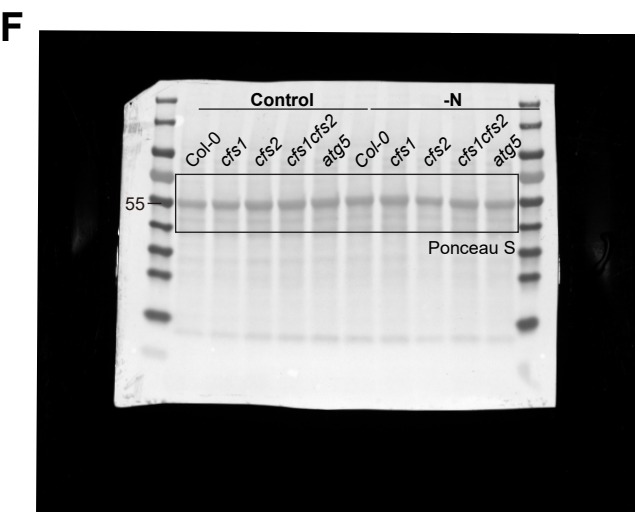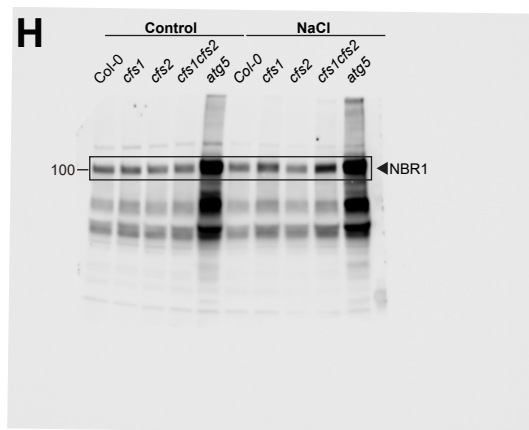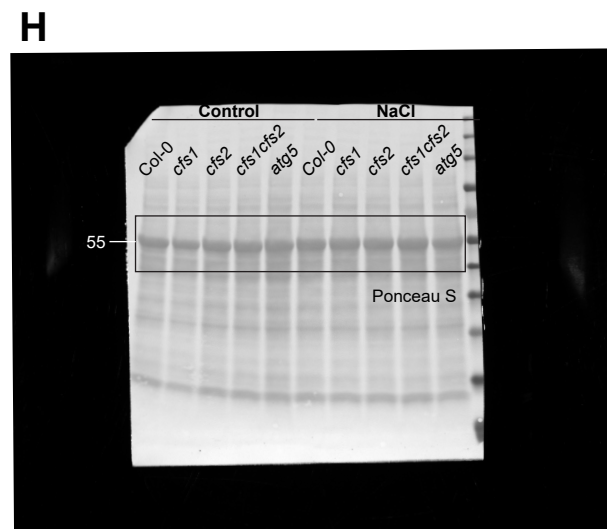

Supplement: SourceData FS2 — is the source file for Fig. S2. [file JCB_202203139_SourceDataFS2.pdf]

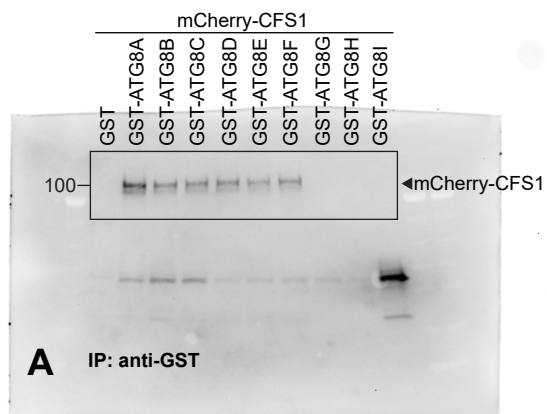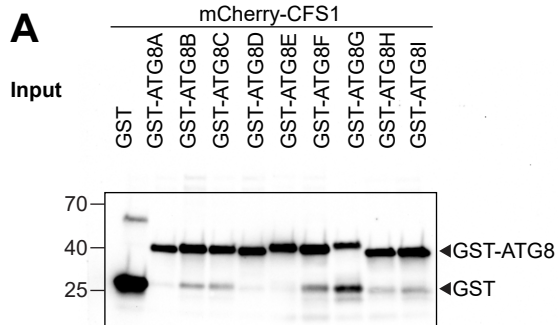

**A** IP: anti-GST

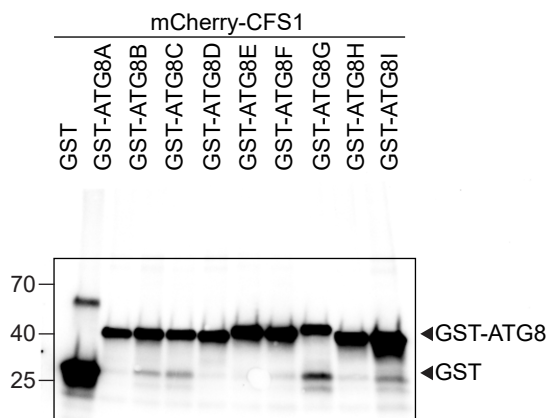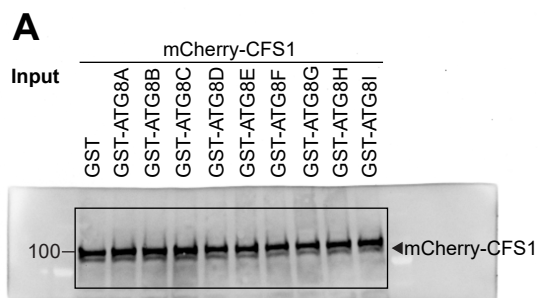

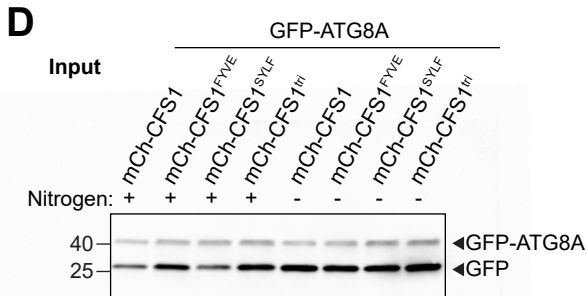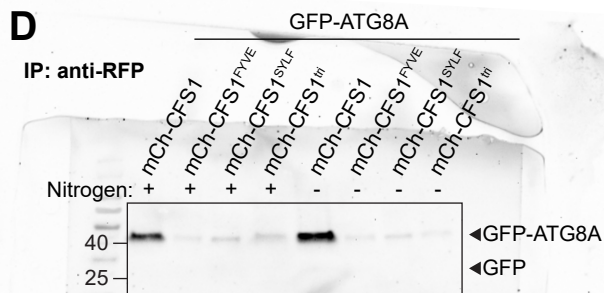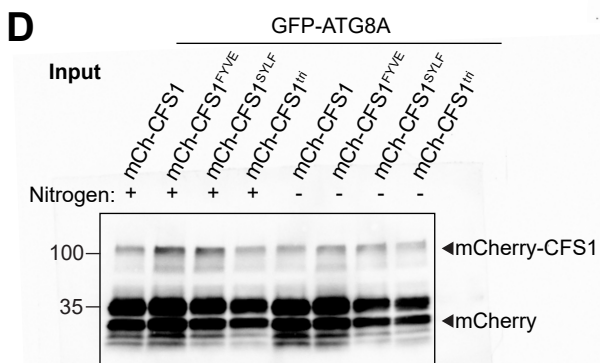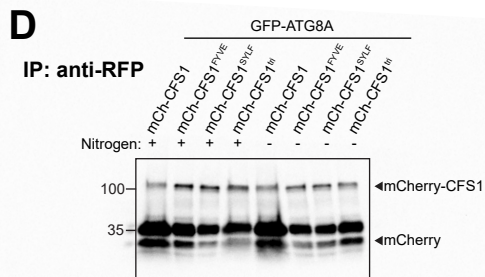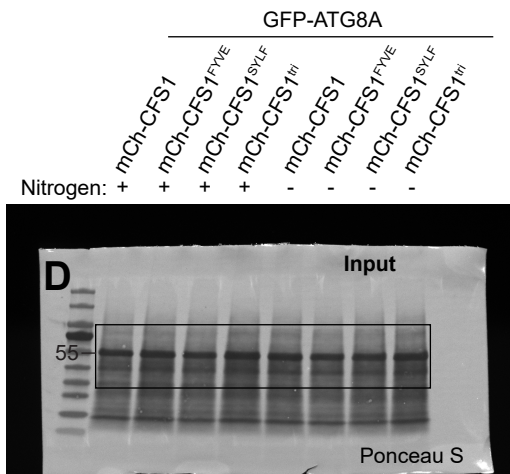

Supplement: SourceData FS3 — is the source file for Fig. S3. [file JCB_202203139_SourceDataFS3.pdf]
